# Fibrostricturing Crohn's Disease Is Marked by an Increase in Active Eosinophils in the Deeper Layers

**DOI:** 10.14309/ctg.0000000000000706

**Published:** 2024-05-01

**Authors:** Inge Jacobs, Bo-Jun Ke, Matthias Ceulemans, Jonathan Cremer, André D'Hoore, Gabriele Bislenghi, Gianluca Matteoli, Gert De Hertogh, João Sabino, Marc Ferrante, Séverine Vermeire, Christine Breynaert, Tim Vanuytsel, Bram Verstockt

**Affiliations:** 1 Katholieke Universiteit Leuven, Department of Microbiology, Immunology and Transplantation, Allergy and Clinical Immunology Research Group, Leuven, Belgium;; 2 Katholieke Universiteit Leuven, Department of Chronic Diseases and Metabolism (ChroMetA), Translational Research Centre for Gastrointestinal Disorders (TARGID), Leuven, Belgium;; 3 University Hospitals Leuven, Department of Gastroenterology and Hepatology, Leuven, Belgium;; 4 Katholieke Universiteit Leuven, Department of Imaging and Pathology, Translational Cell & Tissue Research, Leuven, Belgium;; 5 University Hospitals Leuven, Department of General Internal Medicine, Leuven, Belgium.

**Keywords:** fibrosis, eosinophils, immune landscape, Crohn's disease

## Abstract

**INTRODUCTION:**

Approximately 50% of patients with Crohn's disease (CD) develop intestinal strictures necessitating surgery. The immune cell distribution in these strictures remains uncharacterized. We aimed to identify the immune cells in intestinal strictures of patients with CD.

**METHODS:**

During ileocolonic resections, transmural sections of terminal ileum were sampled from 25 patients with CD and 10 non-inflammatory bowel disease controls. Macroscopically unaffected, fibrostenotic, and inflamed ileum was collected and analyzed for immune cell distribution (flow cytometry) and protein expression. Collagen deposition was assessed through a Masson Trichrome staining. Eosinophil and fibroblast colocalization was assessed through immunohistochemistry.

**RESULTS:**

The Masson Trichrome staining confirmed augmented collagen deposition in both the fibrotic and the inflamed regions, though with a significant increased collagen deposition in the fibrotic compared with inflamed tissue. Distinct Th1, Th2, regulatory T cells, dendritic cells, and monocytes were identified in fibrotic and inflamed CD ileum compared with unaffected ileum of patients with CD as non-inflammatory bowel disease controls. Only minor differences were observed between fibrotic and inflamed tissue, with more active eosinophils in fibrotic deeper layers and increased eosinophil cationic protein expression in inflamed deeper layers. Last, no differences in eosinophil and fibroblast colocalization were observed between the different regions.

**DISCUSSION:**

This study characterized immune cell distribution and protein expression in fibrotic and inflamed ileal tissue of patients with CD. Immunologic, proteomic, and histological data suggest inflammation and fibrosis are intertwined, with a large overlap between both tissue types. However strikingly, we did identify an increased presence of active eosinophils only in the fibrotic deeper layers, suggesting their potential role in fibrosis development.

## INTRODUCTION

Crohn's disease (CD) is characterized by a relapsing-remitting disease course, with transmural intestinal inflammation as a hallmark feature (1). Therefore, patients with CD are at risk of developing (small) bowel strictures for which surgical intervention is required (1). Moreover, stricture recurrence is common and cannot always be prevented, resulting in repetitive resections and the potential risk of developing short bowel syndrome (2).

Research has primarily focused on mucosal inflammation, rather than on the resulting fibrostenosis. Due to continuous intestinal injury, growth factors, such as transforming growth factor β (TGF β), are released, which in turn stimulate fibroblast activation and differentiation toward myofibroblasts (3). Subsequently, excessive extracellular matrix deposition will occur, resulting in fibrosis (4). In the lung, liver, and intestine, it has already been demonstrated that myofibroblasts and active fibroblasts contribute to the development of fibrosis (5–8). However, targeting active fibroblasts and myofibroblasts should be approached with caution, as fibroblasts initiate and propagate wound healing (9). Of importance, there is a strong crosstalk between fibroblasts and various immune cells. Immune cells produce mediators that are capable of activating fibroblasts, while fibroblasts can reciprocally modulate the activity of immune cells (10,11).

Although an involvement of several immune cells such as eosinophils, monocytes, dendritic cells, and T cells has been reported in fibrotic processes in other organs, the key immune cells and their mediators involved in the process of stricturing CD remain poorly understood.

Colon et al (12) have shown that a deletion of eosinophil peroxidase, a mediator solely produced by eosinophils, can decrease renal fibrosis development by decreasing α-smooth muscle actin levels and collagen I deposition in a murine model. In line, anti–interleukin (IL)-5–mediated eosinophil targeting in mice revealed that eosinophils also play a profibrotic role in hepatic fibrosis (13). Increased monocyte counts have previously been linked to worse outcomes in both idiopathic pulmonary and colonic fibrosis (7,14). Similarly, dendritic cells were revealed to contribute to fibrosis by activating myofibroblasts (9). Furthermore, the adaptive immune system has previously been described in fibrotic processes as well. In that context, T helper 2 (Th2) and regulatory T cells (Treg) were reported to have profibrotic characteristics, while Th1 cells rather act as anti-fibrotic cells (15–18).

Although several reports about the involvement of these cells in the development of fibrosis have been published, they largely rely on murine studies and organs outside the gastrointestinal tract. A detailed characterization of the immune cells involved in human fibrostricturing CD is currently lacking. Therefore, we aimed to map the key immune cells contributing to fibrosis in patients with CD.

## MATERIALS AND METHODS

### Patient samples

Resection specimens were prospectively collected through dermal punch biopsy from 25 patients with CD with fibrostricturing disease requiring ileocolonic resection and from 10 non-inflammatory bowel disease (IBD) controls diagnosed with colorectal cancer and in whom a right hemicolectomy was required. From the CD specimens, macroscopically unaffected, inflamed (ulceration), and fibrostenotic tissue (wall thickening, sampled at site of stricture) was obtained by an IBD specialized pathologist (G.D.H.), while in non-IBD controls, tissue was taken from the macroscopically unaffected resection margin at the terminal ileum. Written informed consent was obtained from each patient under an approved protocol by the University Hospitals of Leuven Ethics Committee Review Board (S53684).

### Isolation of mucosal and deeper layer intestinal leukocytes

**Figure 1.**
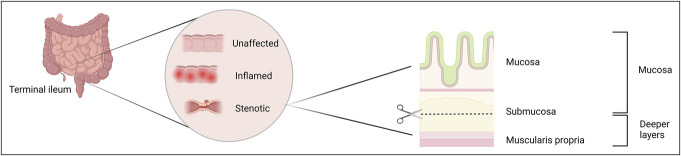
Schematic overview tissue sampling and separation. Samples were taken from unaffected, inflamed, and fibrotic tissue in the terminal ileum of patients with CD, while only unaffected tissue was sampled from non-IBD controls. The tissue was then macroscopically separated into a mucosal region and deeper layers by separating in the submucosa. CD, Crohn's disease; IBD, inflammatory bowel disease.

Mucosal layers were separated macroscopically from deeper layers, and processed within 1 hour of sample collection (Figure 1). The tissue was incubated under magnetic stirring for 10 minutes at 37 °C in RPMI-1640 (Gibco, 21875-034) supplemented with 10% fetal bovine serum (Gibco, 10270-106), 1% penicillin streptomycin 10.000 U/mL (Gibco, 15140-122), 5 mM ethylenediaminetetraacetic acid (Invitrogen, 15575-038), and 2% 1M N-2-hydroxyethylpiperazine-N-2-ethane sulfonic acid (Gibco, 15630-056) to remove mucus and epithelial cells. The epithelial fraction was removed, and this step was repeated once. Afterward, the epithelial fraction was discarded, and the remaining tissue was cut into smaller fractions. The remaining sample was digested using 10 mL Hank's balanced salt solution with Ca^2+^ Mg^2+^ (Gibco, 24020-117) supplemented with 10 mg/mL collagenase type 4 (Worthington, LS004188), 0.2% Deoxyribonuclease I (Roche, 10104159001), and 2% 1M N-2-hydroxyethylpiperazine-N-2-ethane sulfonic acid at 37 °C for 50 minutes to 1 hour. The isolated single-cell fraction was filtered through a 70-μm cell strainer (Greiner Bio-one, 542070), after which the cells were counted and used for flow cytometry.

### Single-cell staining and flow cytometry

Single cells were stained for viability with either Fixable Viability Dye eFluor 450 (Invitrogen, 65-0863-14) or Fixable Viability Dye eFluor 780 (Invitrogen, 65-0865-14) for 25 minutes at room temperature and protected from light. Cells were washed with phosphate-buffered saline (PBS) (Gibco, 14190-094) + 0.5% Bovine Serum Albumin (BSA) (Sigma-Aldrich, 9048-46-8) and centrifuged for 5 minutes at 400 g. The supernatant was discarded, and the pellet was resuspended in blocking mix (2% heat-inactivated plasma in PBS + 0.5% BSA) for 10 minutes at 4 °C. Cells were washed and centrifuged again and incubated in the antibody mixes (see Supplementary Table 1, http://links.lww.com/CTG/B118) for 30 minutes at 4 °C. Afterward, cells were washed again and incubated in 1% PBS-buffered formaldehyde (Merck, 30525-89-4) for 15 minutes at room temperature. Once more, cells were washed with PBS + 0.5% BSA + 2 mM ethylenediaminetetraacetic acid and stored in 4 °C until acquisition.

Samples were acquired on a BD Biosciences (BD) LSR Fortessa Special Order Research Product using BD FACSDiva software version 8. The configuration can be found in Supplementary Digital Content (see Supplementary Table 2, http://links.lww.com/CTG/B118). Quality control was performed before each acquisition by using FACS-Diva CS&T Research Beads (BD, 655051). For fluorescence compensation settings, anti-rat/anti-hamster Ig,κ CompBeads (BD, 552845), anti-mouse Ig,κ CompBeads (BD, 552843), or MACS Comp Beads anti-REA (Miltenyi, 130-104-693) were used. Fluorescence minus one controls were included. Flow cytometry files were analyzed using the BD FlowJo 10 software. Gating strategies can be found in Supplementary Digital Content (see Supplementary Figures 1–3, http://links.lww.com/CTG/B118).

### Protein isolation and measurement

Tissue was mechanically homogenized in PBS + 5% BSA with a Potter-Elvehjem homogenizer after which the homogenized tissue was centrifuged for 5 minutes at 10,000*g*. The supernatant was removed and stored at −80 °C until further analysis.

Eosinophil cationic protein (ECP) was measured through enzyme-linked immunosorbent assay (ELISA) according to the manufacturer's guidelines in duplicate (Abbexa, abx055137). IL-4, IL-5, IL-10, IL-12p70, IL-13, IL-18, basic fibroblast growth factor (bFGF), vascular endothelial growth factor, IL-1β, interferon (IFN)-γ, eotaxin-1,2,3, and TGF-β1,2,3 protein levels were measured with the mesoscale discovery (MSD) U-plex system according to the manufacturer's guidelines (Mesoscale Discovery). All protein levels were corrected for tissue weight.

### Tissue processing and immunohistochemistry

Tissue was processed (see Supplementary Table 3, http://links.lww.com/CTG/B118) and embedded in paraffin after which 5-μm thick slides were cut.

Slides were deparaffinized by incubating them 3 minutes in HistoChoice clearing agent (Sigma Aldrich, H2779) twice, 3 minutes in 50% HistoChoice-ethanol, 3 minutes in 100% ethanol (Merck, 64-17-5) twice, 3 minutes in 95% ethanol, 3 minutes in 70% ethanol, 3 minutes in 50% ethanol, and 3 minutes in demineralized water. Antigen retrieval was performed for 20 minutes at 95 °C using a sodium citrate buffer (10 mM sodium citrate [Merck, 61-32-04-3], 0.05% Tween 20 [Sigma-Aldrich, P9416], pH 9.0). Slides were permeabilized for 10 minutes using 0.3% Triton X-100 (Sigma-Aldrich, 9036-19-5) and 0.3M glycine (Merck, 56-40-6) in PBS after which blocking in 1% BSA in PBS-Tween 20 was performed for 1 hour. Primary antibodies were incubated overnight at 4 °C (see Supplementary Table 4, http://links.lww.com/CTG/B118). The following day, slides were washed 3 times in PBS-Tween 20 for 5 minutes after which secondary antibodies were added for 1 hour at room temperature and protected from light (see Supplementary Table 4, http://links.lww.com/CTG/B118). Slides were washed once for 10 minutes with PBS-Tween 20 after which autofluorescence quenching was performed according to the manufacturer's guidelines (Vector Laboratories, SP-8400-15). 4',6-Diamidino-2-phenylindole was added at room temperature for 15 minutes (see Supplementary Table 4, http://links.lww.com/CTG/B118) after which the slides were washed a last time for 10 minutes with PBS-Tween 20. Finally, the slides were mounted and sealed (Vector Laboratories, SP-8400-15). Images were captured with a 25× water immersion objective (0.8 NA; Zeiss) on an LSM 780 confocal microscope (Carl Zeiss Microscopy). Slides stained for immunohistochemistry were analyzed through ImageJ.

### Evaluation of colocalization

Colocalization was assessed based on the immunohistochemical stainings described earlier. On a random selection of slides (3 patients with CD and 3 non-IBD controls), the shortest distance, determined through a straight line, between eosinophils and (active) fibroblasts was measured through ImageJ. On every slide, 20 shortest distances were measured in the mucosa and 20 shortest distances in the deeper layers, after which the median distance was calculated (19).

### Evaluation of inflammation and fibrosis

Transverse sections (5 μm) were used for a Masson's Trichrome staining, as previously described (20). Collagen area was calculated through ImageJ.

### Statistical analysis

GraphPad prism 9.4.0 was used to perform the statistical analysis. Normality was determined using a Shapiro-Wilk test, after which an unpaired analysis (unpaired *t* test or Mann-Whitney analysis) between the non-IBD controls and patients with CD was performed. Comparisons between tissue layers and regions within the patients with CD were conducted using a Friedmann test. Nonparametric spearman correlations were calculated. Data were represented as median (interquartile range). All analyses were corrected for multiple testing by the Bonferroni method. A corrected *P* value < 0.05 was considered statistically significant.

## RESULTS

### Patient characteristics

Twenty-five patients with CD and 10 non-IBD controls were included. Clinical characteristics are summarized in Table 1.

**Table 1.** Clinical characteristics of patients with CD and non-IBD controls

Clinical features	Patients with CD (n = 25)	Non-IBD controls (n = 10)
Female, n (%)	13 (52)	5 (50)
Median (IQR) age at surgery (yr)	29.5 (25–41.3)	79.0 (54.0–83.0)
Disease duration (IQR) (yr)	8.2 (3–12.5)	
Previous intestinal surgery, n (%)	3 (12)	2 (20)
Disease location		
Ileal (L1), n (%)	16 (64)	
Colonic (L2), n (%)	0 (0)	
Ileo-colonic (L3), n (%)	9 (36)	
Upper GI involvement (L4), n (%)	0 (0)	
Disease behavior		
Inflammatory B1, n (%)	0 (0)	
Stricturing B2, n (%)	17 (68)	
Fistulizing B3, n (%)	8 (32)	
Perianal disease, n (%)	1 (4)	
Smoking status		
Current, n (%)	3 (12)	1 (10)
Previous, n (%)	5 (20)	2 (20)
Never, n (%)	17 (68)	7 (70)
Concomitant medication within 8 wk before surgery		
Biological, n (%)	16 (64)	
Anti-TNF, n (%)	9 (36)	
Anti-IL12/23, n (%)	4 (16)	
Anti-α4β7 integrin, n (%)	3 (12)	
Corticosteroids during surgery, n (%)	2 (8)	

CD, Crohn's disease; IBD, inflammatory bowel disease; IQR, interquartile range; IL, interleukin.

### Assessment of collagen deposition

**Figure 2.**
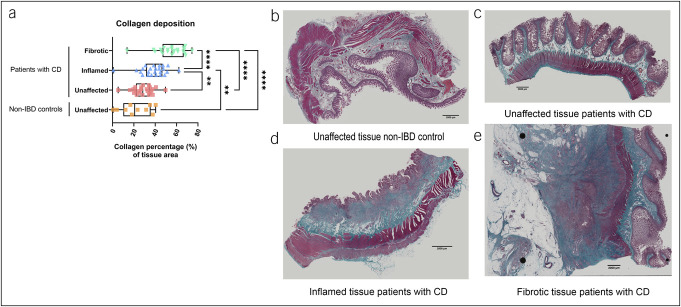
Collagen deposition assessed through Masson's Trichrome staining. Identifying collagen deposition in unaffected, inflamed, and fibrotic regions of non-IBD controls (n = 10) and patients with CD (n = 25) (**a**). Representative slides from the unaffected region of non-IBD controls (**b**) and patients with CD (**c**), inflamed region from patients with CD (**d**), and fibrotic region from patients with CD (**e**) are depicted. Scalebar represents 2000 μm. Data are represented as median + IQR. ***P ≤ 0.001 and ****P ≤ 0.0001. CD, Crohn's disease; IBD, inflammatory bowel disease; IQR, interquartile range.

To morphologically characterize tissue and assess fibrosis formation, collagen deposition was assessed in the unaffected tissue of both non-IBD controls and patients with CD and the inflamed and fibrotic regions of patients with CD. Both inflamed and fibrotic regions exhibited increased collagen deposition in comparison with the unaffected region of the non-IBD controls (*P* = 0.002 and *P* < 0.0001, respectively) and patients with CD (*P* = 0.002 and *P* < 0.0001, respectively). Collagen deposition was more pronounced in the fibrotic region compared with that in the inflamed area (*P* < 0.0001) (Figure 2).

### Immune cell characterization by flow cytometry

To assess the distribution of immune cells in the fibrotic region and in the inflamed tissue of patients with CD, flow cytometry was performed and compared with that in the unaffected tissue of non-IBD controls and the patients with CD.

**Figure 3.**
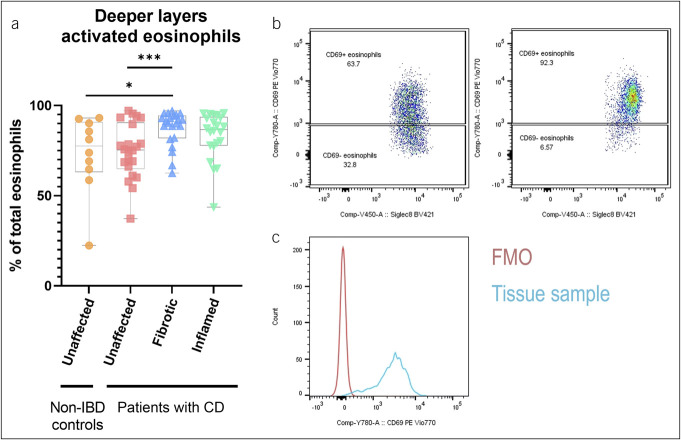
Eosinophil activation in deeper layers. Activation of eosinophils, assessed through the mean fluorescence intensity of CD69, in the deeper layers of non-IBD controls (n = 10) and patients with CD (n = 25) (**a**). Data are represented as median + IQR. Dot plot of single cells isolated from the unaffected deeper layers (left panel) and fibrotic deeper layers (right panel) are shown (**b**). Histogram of the FMO for CD69 PE Vio770 and a representative tissue sample are shown as well (**c**). *P < 0.05, **P ≤ 0.01. CD, Crohn's disease; FMO, fluorescence minus one; IBD, inflammatory bowel disease; IQR, interquartile range.

Although no difference in total eosinophil count was observed, active eosinophils, determined through CD69 expression, were enriched in the fibrotic deeper layers when compared with those in the unaffected deeper layers of patients with CD and non-IBD controls (*P* = 0.0008 and *P* = 0.02) (Figure 3).

Furthermore, the monocyte count was elevated both in the fibrotic (*P* = 0.006 for mucosa and *P* = 0.0004 for deeper layers) and in the inflamed regions (*P* = 0.001 for mucosa and *P* = 0.008 for deeper layers) when compared with the unaffected region in non-IBD controls. In comparison with the unaffected region of patients with CD, monocytes were elevated in the deeper layers only (both *P* < 0.0001 for inflamed and fibrotic regions). Moreover, the total dendritic cell population was increased in both the fibrotic and in the inflamed deeper layers in comparison with the unaffected deeper layers of the patients with CD (both *P* < 0.0001) and non-IBD controls (*P* = 0.002 and *P* = 0.009).

Focusing on the adaptive immune system, Th2 cells were increased in the fibrotic and in the inflamed deeper layers when compared with the unaffected deeper layers of patients with CD (*P* = 0.0002 and *P* < 0.0001) and non-IBD controls (*P* = 0.02 and *P* = 0.04). Mucosal Th2 cells in the fibrotic and in the inflamed region were only increased in comparison with the unaffected mucosa of patients with CD (*P* = 0.008 and *P* = 0.01). Similarly, Treg were increased in both the fibrotic and inflamed mucosa and deeper layers in comparison with the unaffected ileum (*P* < 0.05 for all comparisons). Finally, while we observed a shift toward an increased presence of Th2 cells and Treg, we noticed an opposite trend in Th1 cells, which were decreased in all inflamed and all fibrostenotic layers when compared with paired unaffected regions (*P* < 0.05 for all comparisons).

**Figure 4.**
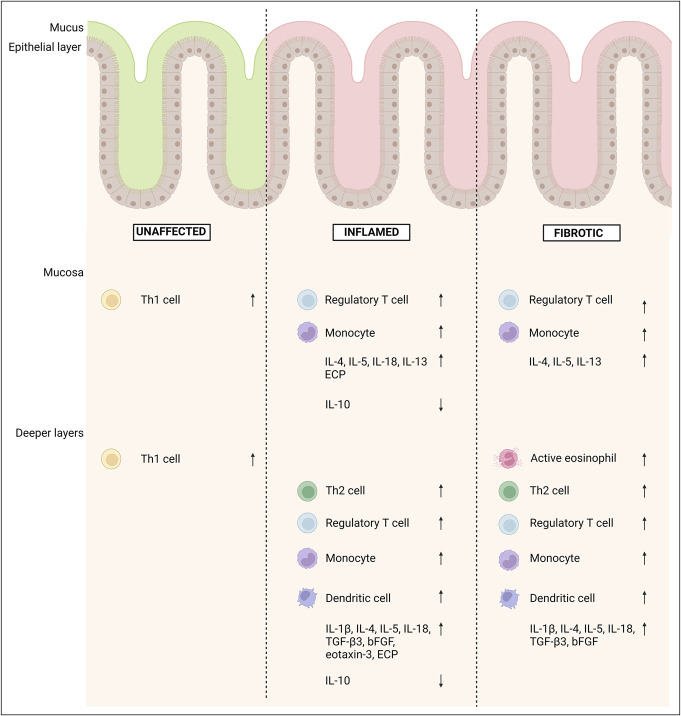
Immune cell distribution and protein expression. Terminal ileal tissue of patients with CD (n = 25) and non-IBD controls (n = 10) was macroscopically separated into mucosa and deeper layers. Immune cell distribution was determined through flow cytometry. Protein expression was determined through MSD and ELISA. Fibrotic and inflamed mucosa and deeper layers were compared with the unaffected area of both patients with CD and non-IBD controls. bFGF, basic fibroblast growth factor; CD, Crohn's disease; IBD, inflammatory bowel disease; IL, interleukin; TGF, transforming growth factor.

No other significant differences in immune cell distribution (Total B cells and T cells, T_FH_, Th9, Th17, Th22, Th1/17, mast cells, neutrophils, and basophils) were detectable in the inflamed and fibrotic regions when compared with the unaffected region. The median (interquartile range) of all immune cells observed through flow cytometry are available in Supplementary Digital Content (see Supplementary Table 5, http://links.lww.com/CTG/B118). Flow cytometry data are summarized in Figure 4 and Supplementary Digital Content (see Supplementary Table 5, http://links.lww.com/CTG/B118).

### Key protein expression levels during fibrosis and inflammation

Because a differential presence of several immune cells was identified through flow cytometry, we additionally studied differences at the protein level using ELISA and the MSD platform. Therefore, our investigation focused on cytokines and proteins synthesized by the immune cells that were associated with inflammation and fibrosis based on flow cytometry.

IL-5 protein expression was significantly upregulated in both the fibrotic and the inflamed regions compared with non-IBD controls (*P* < 0.05 for all comparisons). Likewise, the IL-1β protein expression in inflamed and fibrotic deeper layers was increased in comparison with that in unaffected CD tissue (*P* = 0.01 and *P* = 0.007). Next, IL-4 was significantly increased in the fibrotic and in the inflamed mucosa and deeper layers when compared with the unaffected region in non-IBD controls and patients with CD (*P* < 0.05 for all comparisons). Off note, IL-4 in the deeper fibrotic layers was elevated in comparison with the inflamed deeper layers as well (*P* = 0.03). However, an increased IL-13 expression could only be found in the fibrotic and inflamed mucosa when compared with the unaffected mucosa of non-IBD controls (*P* = 0.007 and *P* = 0.003, respectively). In addition, TGF-β3 protein levels were increased in the fibrotic and in the inflamed deeper layers when compared with non-IBD controls (*P* = 0.007 and *P* = 0.002). IL-18 protein expression was significantly increased in the fibrotic deeper layers (*P* = 0.02), inflamed mucosa (*P* = 0.05), and inflamed deeper layers (*P* = 0.01) in comparison with the non-IBD controls. When compared with the unaffected region of patients with CD, only fibrotic deeper layers and inflamed mucosa showed significantly elevated IL-18 levels (both *P* = 0.007). Last, a similar trend for basic FGF could be observed. In comparison with the deeper layers of the non-IBD controls, significantly increased bFGF protein expression in the fibrotic and in the inflamed deeper layers were found (*P* = 0.02 and *P* = 0.0003). Last, only in the inflamed region, a decreased presence of the anti-inflammatory cytokine IL-10 could be identified (*P* = 0.04 in the mucosa and *P* = 0.006 in the deeper layers).

Overall, protein levels of the selected cytokines were very similar in the fibrotic and in the inflamed tissues. Based on the increased proportion of active eosinophils in the fibrotic regions, we assessed the expression of eosinophil chemokines and granular proteins.

Eotaxin-3 was elevated only in the inflamed deeper layers, when compared with the deeper layers of non-IBD controls (*P* = 0.008). ECP expression levels furthermore were increased in both the inflamed mucosa and deeper layers, in comparison with the non-IBD controls (*P* = 0.004 and *P* = 0.0005) and the CD unaffected area (*P* = 0.04 and *P* = 0.02). No such difference could be found between the fibrostenotic and control regions (*P* = 0.3 for mucosa and *P* = 0.2 for deeper layers). Protein expression levels are summarized in Figure 4 and Supplementary Digital Content (see Supplementary Table 6, http://links.lww.com/CTG/B118).

### Assessing eosinophil and (active) fibroblast colocalization

**Figure 5.**
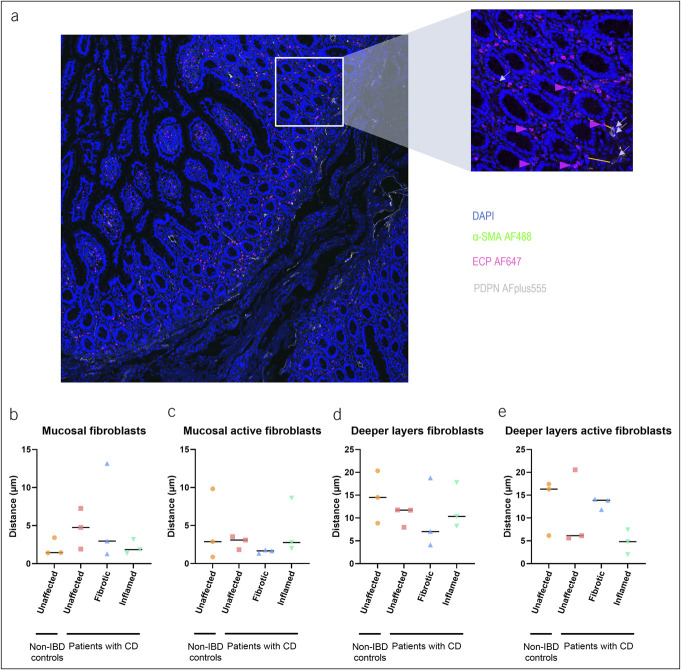
Eosinophil and (active) fibroblast colocalization. Representative immunofluorescence of ECP, podoplanin (PDPN), and α-SMA in the terminal ileum of patients with CD (n = 3) and non-IBD controls (n = 3). Arrowheads indicate eosinophils, and arrows indicate fibroblasts. Shortest distances are represented by an orange straight line (**a**). Unaffected, fibrotic, and inflamed regions were stained. Nuclei were stained with DAPI. Calculated distances between the eosinophils and the fibroblasts in the mucosa (**b**) and deeper layers (**c**) are shown. Distances between the eosinophils and the active fibroblasts in the mucosa (**d**) and deeper layers (**e**) are represented as well. CD, Crohn's disease; IBD, inflammatory bowel disease.

To further examine the potential role of eosinophils in fibrosis and given the established role of fibroblasts in fibrosis, we investigated colocalization of eosinophils and (active) fibroblasts in the fibrotic regions on immunohistochemistry (8,19). Both cell types colocalized in all examined layers, and no difference in colocalization between the fibrotic deeper layers and the unaffected deeper layers was observed (of both patients with CD and non-IBD controls) (Figure 5). Results are summarized in Supplementary Digital Content (see Supplementary Table 7, http://links.lww.com/CTG/B118).

## DISCUSSION

The pathophysiology of CD-related fibrosis is still largely unknown. In this study, we demonstrated a differential distribution of active eosinophils, Th1 cells, Th2 cells, Treg, monocytes, and dendritic cells in the different tissue layers of patients with fibrostenotic CD in comparison with that in control tissue. Strikingly, the immunological landscape between fibrotic and inflamed regions looked largely similar, with activated eosinophils as the only differentiator.

We observed striking similarities between the inflamed and the fibrotic regions for the immune cell composition, protein expression levels, and distances between eosinophils and (active) fibroblasts. In addition, although collagen deposition was more pronounced in the fibrotic region, confirming the clinical categorization of fibrotic vs inflamed region, the inflamed region also showed significantly increased collagen accumulation in comparison with the unaffected tissue. These findings suggest that inflammation and fibrosis are closely intertwined processes, indicating that remodeling might be spread further than what first meets the eye. Previous literature also suggested that fibrosis is unlikely to occur without the presence of inflammation (21), although the question of whether inflammation drives fibrosis is a topic of ongoing debate and remains a central point of interest (21). Nonetheless, in our cohort, we did identify 2 factors that differed between these regions: the presence of active eosinophils and eotaxin-3 and ECP protein expression.

Eosinophils have previously been described to be profibrotic in murine models of renal, pulmonary, hepatic, and radiation-induced fibrosis (9,22–24). However, no such data exist for the human gut. In our cohort of patients with CD undergoing surgery for stricturing disease, we observed an increase in activated eosinophils, but not of the total eosinophil count, in the fibrotic deeper layers. However, eosinophils did not colocalize specifically with active fibroblasts in the fibrostenotic tissue. Furthermore, both in the inflamed and the fibrotic regions, we could observe an increased protein expression of IL-1β, IL-4, IL-5, and bFGF. These proteins can all be produced by the eosinophils and have previously been described to stimulate inflammation and fibrosis (25–28). Through these proteins, eosinophils could potentially mediate the process of inflammation and fibrosis. Of interest, IL-4 was elevated in the fibrotic deeper layers in comparison with the inflamed deeper layers as well and might contribute to the presence of activated eosinophils solely in the fibrotic deeper layers. In contrast to the increased presence of active eosinophils in the fibrotic deeper layers, the inflamed deeper layers embedded a higher concentration of ECP, which is only produced and secreted by eosinophils. Taken together, these findings might suggest that eosinophils do play a role in both the fibrotic and inflammatory processes, but potentially through different mediators in each process. Hence, eosinophils and their signaling molecules should be further explored as potential new therapeutic targets in fibrostenosing CD.

Subsequently, we demonstrated increased Th2 and Treg, but a decrease in the total amount of Th1 cells in both the fibrotic and in the inflamed regions. Th2 cells have previously been proposed as profibrotic and proinflammatory cells through their ability to produce IL-4 and IL-13 (29). In line with this, we similarly observed an increase in IL-4 and IL-13 protein expression in our cohort. Similar as to the eosinophils, Th2 cells can produce the proinflammatory and profibrotic cytokines IL-1β, IL-4, IL-5, and bFGF (25–28,30). Hence, our findings might point toward a proinflammatory and profibrotic role for the Th2 cells, although no causal or mechanistic relationship has ever been described between fibrosis and Th2 immune responses. Consistent with the current findings, Barrow and Wynn (18) previously showed that mice that developed biliary fibrosis expressed increased Th2 and Treg levels. However, the role of Treg in murine fibrosis models is still being debated. These adaptive immune cells have been described as profibrotic, antifibrotic, or to have no role at all in the development of fibrosis (31–33). For example, Treg deletion was shown to reduce cardiac fibrosis and bleomycin-induced lung fibrosis (16). Moreover, previous studies have also indicated a clear Treg plasticity. It has been shown that Treg can ultimately lose their suppressor ability and adopt a Th2 phenotype through which they can promote fibrosis and remodeling and thereby also exacerbate the existing fibrotic response without initiating the response of excessive healing (33,34).

Last, both dendritic cells and monocytes have been described to be profibrotic and proinflammatory in a multitude of studies and were increased in the fibrotic and in the inflamed regions of these resection specimens (19,35–38). Increased monocyte counts have previously been linked to worse outcomes in idiopathic pulmonary fibrosis (14). In addition, a C-C chemokine receptor type 2+ subset contributed to colonic fibrosis in a chronic dextran sodium sulphate colitis model, highlighting a potential profibrotic role (7). Our findings furthermore revealed an increase in IL-18 in the inflamed and in the fibrotic regions. IL-18 has previously been identified to be upregulated in patients with CD and to have a strong proinflammatory and profibrotic role (39–41). In that way, and because monocytes are potent producers of IL-18, these cells could facilitate inflammation and fibrosis. Similarly, dendritic cells have been shown to contribute to fibrosis by activating myofibroblasts (9). These cell types were found to be elevated in the inflamed and fibrotic regions in our cohort, supporting a role in fibrosis and inflammation.

Previously, several studies have investigated the coculturing of eosinophils and fibroblasts. In a first study, IL-33–primed eosinophils were cocultured with intestinal fibroblasts, leading to the release of various profibrotic factors such as IL-1β, IL-6, and periostin (42). More recently, Kuwabara et al (43) demonstrated that human eosinophils could enhance α-smooth muscle actin expression in a human fetal lung fibroblast cell line when cocultured, indicating a shift toward active fibroblasts. In addition, the impact of IL-3–activated eosinophils on human lung fibroblasts was examined by exposing the fibroblasts to eosinophil degranulating products, resulting in significant changes in gene expression and thereby underscoring the potential importance of eosinophil-fibroblast interactions in tissue remodeling (44).

Although this is the first study profiling the immunological landscape in fibrostricturing CD using flow cytometry, the current design allowed only for association, but cannot establish a causal relationship. Without additional single-cell analyses or a further immunological characterization by flow cytometry, we cannot infer about more specific subpopulations of the various immune cell subtypes. However, identifying eosinophils through single-cell sequencing has shown to be challenging due to their high ribonuclease content, and therefore, flow cytometry still has its place in the immunoprofiling of patients with IBD. Similarly, low sample sizes used in single-cell sequencing assays can be problematic when studying a heterogenous disease such as CD. Nonetheless, further research using novel techniques could lead to a better understanding of our findings through more advanced cellular characterization. In that way, we could obtain better insights on the functionality of these immune cells.

In conclusion, we studied transmural intestinal sections of a unique cohort of patients with CD undergoing surgery for a fibrostenotic disease. We were able to identify the differential immune cell distribution and protein expression in unaffected, inflammatory, and fibrotic terminal ileum. Activated eosinophils were more abundant in the deeper layers of the fibrotic tissues, highlighting them and their secreted products as potential treatment targets in fibrostenotic CD.

## CONFLICTS OF INTEREST

**Guarantor of the article:** Bram Verstockt, MD, PhD.

**Specific author contributions:** I.J., J.S., M.F., S.V., C.B., T.V., B.V.: study concept and design. A.D., G.B.: intestinal tissue sampling. G.D.H.: histological scoring. I.J., B.J.K., J.C.: data acquisition and analysis. I.J., B.J.K., J.C., G.M., J.S., M.F., S.V., C.B., T.V., B.V.: interpretation of data. I.J.: writing the initial manuscript. All authors approved the final version of the manuscript.

**Financial support:** This research has been funded by a Beligan Inflammatory Bowel Disease Research and Development (BIRD) grant and an internal C1 project (ZKD2906 – C14/17/097) of Katholieke Universiteit Leuven and BIRD grant. T.V., M.F., and J.S. are supported by a senior clinical research fellowship of the Flanders Research Foundation (FWO Vlaanderen; T.V.: 1830517N). B.V. and C.B. are supported by the Clinical Research Fund (KOOR) at the University Hospitals Leuven. B.V. is also supported by the Research Council at the Katholieke Universiteit Leuven. S.V. holds a BOF-FKO from the Katholieke Universiteit Leuven.

**Potential competing interests:** T.V. has received research support and lecture and consultancy fees from Takeda. B.V. reports financial support for research from AbbVie, Biora Therapeutics, Landos, Pfizer, Sossei Heptares, and Takeda; lecture fees from Abbvie, Biogen, Bristol Myers Squibb, Celltrion, Chiesi, Falk, Ferring, Galapagos, Janssen, Lily, MSD, Pfizer, R-Biopharm, Takeda, Truvion, and Viatris; and consultancy fees from Abbvie, Alimentiv, Applied Strategic, Atheneum, Biora Therapeutics, Bristol Myers Squibb, Galapagos, Guidepont, Mylan, Inotrem, Ipsos, Janssen, Lily, Progenity, Sandoz, Santa Ana Bio, Sosei Heptares, Takeda, Tillots Pharma, and Viatris. M.F. receives financial support for research from AbbVie, Amgen, Biogen, EG, Janssen, Pfizer, Takeda, and Viatris; receives speakers' fees from AbbVie, Amgen, Biogen, Boehringer Ingelheim, Falk, Ferring, Janssen-Cilag, Lamepro, MSD, Pfizer, Sandoz, Takeda, Truvion Healthcare, and Viatris; and does consultancy for AbbVie, AgomAb Therapeutics, Boehringer Ingelheim, Celgene, Celltrion, Eli Lilly, Janssen-Cilag, Medtronic, MSD, Pfizer, Regeneron, Samsung Bioepis, Sandoz, Takeda, and ThermoFisher. J.S. receives financial support for research from Galapagos and Viatris; receives speakers' fees from Abbvie, Falk, Takeda, Pfizer, Galapagos, Ferring, Janssen, and Fresenius; and does consultancy for Janssen, Ferring, Fresenius, Abbvie, Galapagos, Celltrion, Pharmacosmos, and Pharmanovia. S.V. receives financial support for research from AbbVie, J&J, Pfizer, Takeda, and Galapagos and receives speakers' and consultancy fees from AbbVie, AbolerIS Pharma, AgomAb, Alimentiv, Arena Pharmaceuticals, AstraZeneca, Avaxia, BMS, Boehringer Ingelheim, Celgene, CVasThera, Cytoki Pharma, Dr Falk Pharma, Ferring, Galapagos, Genentech-Roche, Gilead, GSK, Hospira, Imidomics, Janssen, J&J, Lilly, Materia Prima, MiroBio, Morphic, MrMHealth, Mundipharma, MSD, Pfizer, Prodigest, Progenity, Prometheus, Robarts Clinical Trials, Second Genome, Shire, Surrozen, Takeda, Theravance, Tillots Pharma AG, and Zealand Pharma. G.D.H. receives fees for his activities as central pathology reader from Centocor and receives speakers' fees from Janssen. G.B. receives speakers' fees from Galapagos, Janssen, and Takeda. C.B. has received research fees from Ablynx.

**Data availability statement:** The data underlying this article will be shared on reasonable request.

**Study Highlights**

**WHAT IS KNOWN**

✓ Patients with Crohn's Disease (CD) often develop strictures.
✓ The immune landscape involved in the process of the development of strictures is poorly understood.

**WHAT IS NEW HERE**

✓ We identified the immune landscape in fibrostenotic obstructions of patients with CD.
✓ We primarily identified an increase in active eosinophils in the fibrotic deeper layers.
✓ This immunological characterization can aid to prioritize possible antifibrotic targets for stricture development in patients with CD.

## Supplementary Material
