# Supplementary material for: Fibrostricturing Crohn's Disease Is Marked by an Increase in Active Eosinophils in the Deeper Layers
Source: Clin Transl Gastroenterol. 2024 May 1;15(7):e00706. doi: 10.14309/ctg.0000000000000706 (PMC11272291; doi:10.14309/ctg.0000000000000706)
Supplement: Supplementary file 1 [file ct9-15-e00706-s001.docx]

***Supplementary table 1:*** Overview of antibodies used to detect immune cells

Fixable Viability Dye eFluor™ (FVD), Cluster of differentiation (CD), Brilliant Violet™ (BV), Alexa Fluor® (AF), Allophycocyanin (APC), Peridinin Chlorophyll Protein Complex (PerCP), Phycoerythrin (PE), Super Bright (SB), Fluorescein Isothiocyanate (FITC)

| **Target** | **Clone** | **Fluorophore** | **Company** | **Catalogue number** | **Isotype** |
| --- | --- | --- | --- | --- | --- |
| *Panel 1* | | | | | |
| **Viability** |  | FVD450 | eBioscience | 65-0863-18 | |
| **CD25** | 2A3 | BV711 | BD | 563159 | Mouse IgG1, k |
| **CD183** | 1C6/CXCR3 | AF488 | BD | 558047 | Mouse IgG1, k |
| **CD127** | eBioRDR5 | APC-eFluor780 | eBioscience | 47-1278 | Mouse IgG1, k |
| **CD161** | HP-3G10 | PerCP-Cy5.5 | Biolegend | 339908 | Mouse IgG1, k |
| **CD197** | 150503 | PE-CF594 | BD | 562381 | Mouse IgG2a, k |
| **CD3** | UCHT1 | APC-R700 | BD | 565119 | Mouse IgG1, k |
| **CD194** | 1G1 | PE | BD | 551120 | Mouse IgG1, k |
| **CD196** | G034E3 | BV785 | Biolegend | 353422 | Mouse IgG2b, k |
| **CD185** | RF8B2 | Biotin | BD | 552118 | Rat IgG2b, k |
| **Streptavidin** |  | PE-Cy7 | BD | 557598 |  |
| **CD4** | SK3 | BV510 | BD | 562970 | Mouse IgG1, k |
| **CD45RA** | HI100 | BV570 | Biolegend | 304132 | Mouse IgG2b, k |
| **CCR10** | 1B5 | APC | BD | 564771 | Mouse IgG2a, k |
| **CD20** | 2H7 | PE-Cy5.5 | eBioscience | 35-0209 | Mouse IgG2b, k |
| **CD8a** | RPA-T8 | SB645 | eBioscience | 64-0088 | Mouse IgG1, k |
| **Brilliant stain buffer** | | | BD | 566385 | |
| *Panel 2* | | | | | |
| **Viability** |  | FVD780 | eBioscience | 65-0865-18 | |
| **CD125** | A14 | PE | BD | 555902 | Mouse IgG1, k |
| **Siglec-8** | 837535 | BV421 | BD | 747875 | Mouse IgG1,k |
| **CD123** | 6H6 | FITC | Biolegend | 306014 | Mouse IgG1,k |
| **CD45** | 2D1 | AF700 | eBioscience | 56-9459-42 | Mouse IgG1, k |
| **CD69** | REA824 | PE-Vio770 | Miltenyi | 130-112-615 | Recombinant |
| **CD193** | 5E8 | APC | Biolegend | 310708 | Mouse IgG2b, k |
| **CD117** | 104D2 | BV711 | Biolegend | 313230 | Mouse IgG1, k |
| **CD16** | 3G8 | PerCP-Cy5.5 | Biolegend | 302028 | Mouse IgG1,k |
| **Brilliant stain buffer** | | | BD | 566385 | |
| *Panel 3* | | | | | |
| **Viability** |  | FVD780 | eBioscience | 65-0865-18 | |
| **Siglec-8** | 7C9 | PE-Dazzle 594 | Biolegend | 347109 | Mouse IgG1,k |
| **CD14** | HCD14 | PE | Biolegend | 325606 | Mouse IgG1,k |
| **CD45** | 2D1 | AF700 | eBioscience | 56-9459-42 | Mouse IgG1, k |
| **CD117** | 104D2 | APC | Biolegend | 313205 | Mouse IgG1, k |
| **CD3** | UCHT1 | FITC | Invitrogen | 11-0038-42 | Mouse IgG1,k |
| **CD19** | HIB19 | FITC | Invitrogen | 11-0199-42 | Mouse IgG1,k |
| **CD56** | 5.1H11 | FITC | Biolegend | 362545 | Mouse IgG1,k |
| **CD206** | 15-2 | PerCP-Cy5.5 | Biolegend | 321121 | Mouse IgG1,k |
| **CD123** | 6H6 | BV650 | Biolegend | 306019 | Mouse IgG1, k |
| **CD11c** | 3.9 | BV421 | Biolegend | 301627 | Mouse IgG1, k |
| **HLA-DR** | L243 | PE-Cy7 | Biolegend | 307615 | Mouse IgG2a, k |
| **CD16** | 3G8 | BV711 | Biolegend | 302043 | Mouse IgG1, k |
| **Brilliant stain buffer** | | | BD | 566385 | |

***Supplementary table 2:*** *configuration flow cytometer*

| **Laser wavelength (nm)** | **Laser Power (mW)** | **Detector** | **Spectral range (nm)** | **Dichroic LP Filter (nm)** | **Band Pass Filter (nm)** | **Fluorochrome detected** |
| --- | --- | --- | --- | --- | --- | --- |
| Flow cytometer configuration for panel 1 | | | | | | |
| 405 (violet) | 50 | \| V785 \| \| --- \| | 755-815 | 735 | 785/60 | BV785 |
|  |  | V711 | 698.5-723.5 | 685 | 711/25 | BV711 |
|  |  | V661 | 651-671 | 630 | 661/20 | SB-645 |
|  |  | V610 | 571-601 | 570 | 586/15 | BV570 |
|  |  | V525 | 505-535 | 505 | 525/50 | BV510 |
|  |  | V450 | 425-475 | - | 450/50 | FVD450 |
| 488 (blue) | 50 | B710 | 685-735 | 685 | 710/50 | PerCP-Cy5.5 |
|  |  | B530 | 515-545 | 505 | 530/30 | AF488 |
|  |  | SSC | 483-493 | - | 488/10 | Side scatter |
| 561 (yellow-green) | 50 | Y780 | 750-810 | 750 | 780/60 | PE-Cy7 |
|  |  | Y710 | 685-735 | 685 | 710/50 | PE-Cy5.5 |
|  |  | Y670 | 655-685 | 635 | 670/30 |  |
|  |  | Y610 | 600-620 | 600 | 610/20 | PE-CF594 |
|  |  | Y585 | 578-592 | - | 585/15 | PE |
| 640 (red) | 40 | R780 | 750-810 | 750 | 780/60 | APC-eFluor780 |
|  |  | R730 | 708-750 | 690 | 730/45 | APC-R700 |
|  |  | R670 | 663-677 | - | 670/14 | APC |
| Flow cytometer configuration for panel 2,3 and 4 | | | | | | |
| 405 (violet) | 50 | \| V785 \| \| --- \| | 755-815 | 735 | 785/60 |  |
|  |  | V711 | 698.5-723.5 | 685 | 711/25 | BV711 |
|  |  | V661 | 651-671 | 630 | 661/20 |  |
|  |  | V610 | 600-620 | 600 | 610/20 |  |
|  |  | V525 | 505-535 | 505 | 525/50 |  |
|  |  | V450 | 425-475 | - | 450/50 | BV421 |
| 488 (blue) | 50 | B710 | 685-735 | 685 | 710/50 | PerCP-Cy5.5 |
|  |  | B530 | 515-545 | 505 | 530/30 | FITC |
|  |  | SSC | 483-493 | - | 488/10 | Side scatter |
| 561 (yellow-green) | 50 | Y780 | 750-810 | 750 | 780/60 | PE-Vio770 |
|  |  | Y10 | 685-735 | 685 | 710/50 |  |
|  |  | Y670 | 655-685 | 635 | 670/30 |  |
|  |  | Y610 | 600-620 | 600 | 610/20 |  |
|  |  | Y585 | 578-592 | - | 585/15 | PE |
| 640 (red) | 40 | R780 | 750-810 | 750 | 780/60 | FVD780 |
|  |  | R730 | 708-750 | 690 | 730/45 | AF700 |
|  |  | R670 | 663-677 | - | 670/14 | APC |

***Supplementary table 3:*** *tissue processing for infiltration with paraffin*

| **Solution** | **Duration** |
| --- | --- |
| 70% ethanol | 1 hour |
| 80% ethanol | 1 hour |
| 99% ethanol | 1 hour |
| 99% ethanol | 1 hour |
| 100% ethanol | 2 hours |
| 100% ethanol | 2 hours |
| 100% ethanol/xyleen | 2 hours |
| Xyleen | 2 hours |
| Xyleen | 3 hours |
| Empty | 15 minutes |
| Paraffin | 3 hours |
| Paraffin | 3 hours |

***Supplementary table 4*** *overview of antibodies used for immunohistochemical stainings*

| **Antibody** | **Company** | **Catalogus number** | **Dilution** |
| --- | --- | --- | --- |
| α-SMA (goat) | Novus Biologicals | NB300-978 | 1:200 |
| Podoplanin NZ-1.3 (rat) | Thermofisher | 14-9381-82 | 1:200 |
| Eosinophil cationic protein EG2 (mouse) | Diagnostics Development | EG2 | 1:200 |
| DAPI | Invitrogen | D1306 | 1:500 |
| Donkey anti-mouse AF647 | Invitrogen | A-31571 | 1:200 |
| Donkey anti-rat AFplus555 | Invitrogen | A-48270 | 1:200 |
| Donkey anti-goat AF488 | Invitrogen | A-11045 | 1:200 |

***Supplementary table 5****: Median (IQR) of the cells identified via flow cytometry*

|  | Unaffected  Mucosa | Unaffected deeper  Layers | unaffected mucosa | Unaffected deeper layers | fibrotic mucosa | Fibrotic deeper layers | inflamed mucosa | inflamed deeper layers |
| --- | --- | --- | --- | --- | --- | --- | --- | --- |
|  | Non-IBD control | | CD patient | | | | | |
| B cells (% of viable cells) | 24.4 (2.4;30.6) | 13.2 (1.0;22.2) | 4.4 (1.9;9.8) | 0.6 (0.1;3.8) | 6.7 (2.5;17.5) | 10.0 (3.3;21.5) | 7.1 (1.5;27.7) | 8.3 (4.3;24.8) |
| T cells (% of viable cells) | 29.0 (16.4;42.5) | 22.2 (3.2;33.6) | 36.5 (31;42.6) | 9.0 (3.7;20.6) | 33.5 (25.2;37.4) | 25.2 (15.2;31.3) | 28.2 (22.1;35.8) | 24.4 (19.1;38.9) |
| Regulatory T cells (% of CD20- CD3+ cells) | 3.5 (2.3;5.8) | 5.8 (5.2;6.9) | 4.1 (2.1;6.6) | 4.6 (2.8;8.0) | 10.1 (7.2;15.2) | 9.5 (6.5;12.2) | 12.9 (9.1;16.5) | 11.9 (9.1;13.6) |
| Naïve regulatory T cells (% of CD20- CD3+ cells) | 0.3 (0.08;0.6) | 0.5 (0.2;0.8) | 0.1 (0.05;0.4) | 0.1 (0.06;0.5) | 0.3 (0.07;1.4) | 1.0 (0.6;1.6) | 0.3 (0.07;1) | 0.6 (0.2;1.4) |
| Memory regulatory T cells (% of CD20- CD3+ cells) | 3.1 (2.2;4.1) | 5.4 (4.8;6.3) | 3.9 (1.8;6.1) | 4.2 (2.5;6.5) | 9.7 (6.3;15.1) | 8.2 (6.2;10.7) | 11.0 (7.8;16.2) | 10.0 (7.5;13.1) |
| T_FH_ (% of CD20- CD3+ cells) | 0.05 (0.01;0.6) | 0.1 (0.04;0.2) | 0.01 (0.008;0.05) | 0.02 (0.006;0.08) | 0.02 (0.02;0.07) | 0.01 (0.008;0.06) | 0.04 (0.02;0.07) | 0.02 (0.01;0.04) |
| Th1 (% of CD20- CD3+ cells) | 11.0 (1.2;24.2) | 11.7 (4.9;18.8) | 3.0 (1.6;11.6) | 7.5 (2.1;12.2) | 1.6 (0.3;3.0) | 3.1 (1.2;5.8) | 0.8 (0.3;2.6) | 1.6 (0.9;8.4) |
| Th2 (% of CD20- CD3+ cells) | 2.5 (0.2;3.0) | 1.5 (0.5;3.0) | 2.3 (1.1;4.0) | 2.8 (1.7;3.4) | 4.8 (2.7;7.4) | 6.1 (3.5;9.2) | 4.7 (1.6;6.3) | 4.6 (2.4;7.9) |
| Th9 (% of CD20- CD3+ cells) | 0.6 (0.5;3.8) | 1.0 (0.9;3.6) | 0.5 (0.4;0.8) | 1.5 (0.8;2.9) | 0.8 (0.3;1.2) | 1.3 (0.8;1.8) | 0.7 (0.5;1.5) | 1.2 (0.5;1.7) |
| Th17 (% of CD20- CD3+ cells) | 0.009 (0.006;0.1) | 0.007 (0.007;0.03) | 0.005 (0.001;0.01) | 0.03 (0.004;0.1) | 0.02 (0.006;0.04) | 0.08 (0.04;0.1) | 0.02 (0.007;0.04) | 0.06 (0.01;0.1) |
| Th22 (% of CD20- CD3+ cells) | 0.01 (0.003;0.06) | 0.03 (0.004;0.05) | 0.02 (0.01;0.03) | 0.05 (0.02;0.2) | 0.06 (0.02;0.1) | 0.07 (0.03;0.1) | 0.04 (0.02;0.06) | 0.09 (0.02;0.2) |
| Th1_17 (% of CD20- CD3+ cells) | 0.3 (0.2;1.3) | 0.3 (0.2;1.2) | 0.2 (0.1,0.2) | 0.3 (0.2;0.7) | 0.2 (0.07;0.3) | 0.3 (0.1;0.4) | 0.2 (0.1; 0.3) | 0.2 (0.1;0.5) |
| Dendritic cells (% of viable cells) | 2.7 (1.3;3.4) | 1.2 (0.1;1.9) | 1.3 (1.1;1.9) | 0.4 (0.2;0.9) | 2.0 (1.1;3.0) | 2.4 (1.5;4.0) | 1.9 (1.0;4.1) | 2.1 (1.1;5.0) |
| Monocytes (% of viable cells) | 0.3 (0.1;0.4) | 0.3 (0.2;0.4) | 0.4 (0.3;0.6) | 0.3 (0.2;0.6) | 0.6 (0.5;0.8) | 0.7 (0.3;1.1) | 0.8 (0.6;1.5) | 0.7 (0.4;1.3) |
| Mast cell (% of CD45+ cells) | 2.4 (0.7;3.9) | 4.9 (2.7;8.1) | 6.2 (4.6;9.2) | 11.2 (7.8;17.0) | 3.3 (2.1;5.5) | 4.3 (2.4;9.4) | 2.5 (0.7;4.7) | 3.4 (1.3;5.4) |
| Neutrophil (% of CD45+ cells) | 0.6 (0.3;0.8) | 5.1 (1.4;16.4) | 1.4 (0.7;3.9) | 9.7 (3.3;15.8) | 5.8 (2.1;14.0) | 3.9 (2.3;10.5) | 9.6 (3.7;16.7) | 7.0 (3.7;12.5) |
| Basophil (% of CD45+ cells) | 0.1 (0.07;0.2) | 0.1 (0.06;0.3) | 0.3 (0.1;0.5) | 0.5 (0.3;1.0) | 0.6 (0.3;0.8) | 0.5 (0.3;0.9) | 0.6 (0.4;1.2) | 0.5 (0.3;1.0) |
| Eosinophil (% of CD45+ cells) | 1.6 (0.5;1.9) | 0.6 (0.3;1.4) | 3.2 (2.2;5.0) | 1.5 (0.7;1.9) | 3.7 (1.1;4.8) | 1.3 (0.4;2.6) | 3.0 (0.9;4.7) | 1.1 (0.5;2.1) |
| CD69+ eosinophils (% of total eosinophils) | 88.9 (84.9;93.8) | 77.6 (63.1;90.8) | 88.3 (79.1;93.2) | 75.4 (64.9;90.6) | 89.7 (80.5;93.9) | 90.1 (81.9;94.5) | 87.2 (77.6;93.1) | 86.7 (77.8;93.6) |
| CD125+ eosinophil (% of total eosinophils) | 9.5 (4.1;30.7) | 7.1 (5.4;25.6) | 6.0 (3.1;13.4) | 11.4 (5.2;36.9) | 7.1 (3.7;25.8) | 6.8 (3.8;17.5) | 6.4 (2.9;11.8) | 7.4 (3.3;13.6) |
| CD193+ eosinophil (% of total eosinophils) | 95.9 (92.2;98.2) | 96.6 (93.2;97.7) | 97.0 (94.1;98.3) | 96.7 (94.0;98.1) | 95.5 (93.7;98.4) | 95.5 (93.0;97.5) | 95.8 (94.5;96.8) | 93.9 (90.9;97.5) |

***Supplementary table 6:*** *Median (IQR) protein expression identified via MSD and ELISA and corrected for weight (in pg/mL/mg)*

|  | Unaffected  Mucosa | Unaffected deeper  Layers | unaffected mucosa | Unaffected deeper layers | fibrotic mucosa | Fibrotic deeper layers | inflamed mucosa | inflamed deeper layers |
| --- | --- | --- | --- | --- | --- | --- | --- | --- |
|  | Non-IBD control | | CD patient | | | | | |
| IL-4 | 0.008 (0.005;0.01) | 0.006 (0.003;0.008) | 0.01 (0.006;0.02) | 0.009 (0.004;0.02) | 0.1 (0.01;3.1) | 0.08 (0.01;4.2) | 0.04 (0.02;0.09) | 0.01 (0.009;0.05) |
| IL-5 | 0.2 (0.1;0.3) | 0.1 (0.03;0.3) | 0.3 (0.2;0.5) | 0.7 (0.1;1.1) | 0.3 (0.1;2.8) | 0.7 (0.4;1.2) | 2.1 (0.5;3.5) | 0.8 (0.5;1.5) |
| IL-10 | 0.08 (0.05;0.1) | 0.02 (0.009;0.07) | 0.1 (0.06;0.2) | 0.03 (0.01;0.08) | 0.1 (0.05;0.2) | 0.1 (0.03;0.3) | 0.2 (0.06;1.1) | 0.1 (0.06;0.5) |
| IL-12p70 | 0.02 (0.01;0.04) | 0.02 (0.02;0.04) | 0.04 (0.02;0.3) | 0.03 (0.03;0.05) | 0.1 (0.04;0.7) | 0.03 (0.02;0.05) | 0.1 (0.05;0.4) | 0.06 (0.04;0.2) |
| IL-13 | 0.1 (0.07;0.1) | 0.2 (0.02;0.2) | 0.1 (0.09;0.3) | 0.2 (0.05;0.4) | 0.2 (0.2;0.6) | 0.1 (0.1;0.2) | 0.6 (0.2;1.1) | 0.3 (0.2;4.3) |
| IL-18 | 236.1 (189.2;282.1) | 22.5 (11.7;124.1) | 433.1 (300.4;650.1) | 44.7 (28.9;64.5) | 173.1 (99.4;385.9) | 90.1 (44.9;137.5) | 162.2 (120.3;224.5) | 91.2 (37.5;208.5) |
| bFGF | 7.7 (5.1;11.3) | 8.5 (7.1;12.1) | 10.3 (4.6;16.7) | 11.3 (9.1;16.8) | 7.7 (4.3;12.9) | 15.8 (9.0;25.6) | 17.0 (5.1;25.0) | 16.1 (11.9;24.3) |
| VEGF | 56.7 (30.8;94.3) | 9.1 (7.4;17.5) | 82.3 (24.3;126.0) | 15.0 (7.0;22.6) | 37.7 (10.1;83.2) | 13.0 (3.6;24.1) | 62.0 (12.4;82.3) | 16.3 (6.4;42.3) |
| IL-1β | 1.5 (0.6;3.0) | 0.6 (0.4;3.0) | 3.9 (1.8;9.7) | 2.0 (0.7;4.2) | 20.1 (6.6;51.1) | 4.4 (3.1;20.0) | 17.7 (5.2;111.2) | 9.7 (3.0;61.7) |
| IFN-γ | 0.4 (0.2;0.7) | 0.2 (0.1;0.5) | 1.8 (1.0;6.1) | 1.0 (0.2;1.6) | 4.7 (2.0;16.6) | 4.9 (1.3;15.6) | 13.6 (4.8;42.3) | 5.1 (2.0;23.4) |
| Eotaxin-1 | 3.8 (1.4;7.5) | 2.4 (1.6;8.1) | 3.2 (1.1;9.6) | 3.6 (1.2;12.9) | 4.1 (2.3;7.9) | 6.2 (1.7;16.5) | 3.8 (0.5;5.5) | 8.0 (5.0;14.7) |
| Eotaxin-2 | Above detection limit | | | | | | | |
| Eotaxin-3 | 1.4 (0.4;4.4) | 0.7 (0.4;1.2) | 0.7 (0.4;1.7) | 2.9 (0.6;5.9) | 1.7 (1.3;11.4) | 1.9 (0.5;8.8) | 3.6 (1.2;15.3) | 4.9 (1.4;17.7) |
| TGF-β1 | 22.4 (19.1;30.8) | 20.2 (12.2;37.5) | 35.9 (25.5;54.7) | 28.0 (18.4;40.6) | 52.2 (28.3;92.3) | 26.7 (15.5;43.5) | 112.3 (43.3;241.6) | 29.9 (18.2;117.6) |
| TGF-β2 | 3.3 (1.9;4.4) | 4.2 (3.3;10.2) | 6.6 (4.9;7.9) | 11.8 (8.6;16.6) | 8.2 (4.9;12.6) | 9.9 (7.2;16.7) | 11.0 (4.3;20.0) | 12.6 (8.1;35.8) |
| TGF-β3 | 0.4 (0.2;0.6) | 0.4 (0.3;0.9) | 0.6 (0.3;0.8) | 0.7 (0.5;1.3) | 0.4 (0.3;1.4) | 2.1 (0.5;3.3) | 1.5 (0.3;4.0) | 2.6 (1.1;6.7) |
| ECP | 27.0 (19.1;39.4) | 17.2 (12.3;24.8) | 33.7 (22.2;55.4) | 15.9 (9.4;27.6) | 42.5 (17.2;75.4) | 40.8 (16.6;60.1) | 73.9 (34.0;202.5) | 52.3 (33.0;75.6) |

***Supplementary table 7:*** *Median distance between eosinophils and (active) fibroblasts*

|  | Unaffected  Mucosa | Unaffected deeper  Layers | unaffected mucosa | Unaffected deeper layers | fibrotic mucosa | Fibrotic deeper layers | inflamed mucosa | inflamed deeper layers |
| --- | --- | --- | --- | --- | --- | --- | --- | --- |
|  | Non-IBD control | | CD patient | | | | | |
| Distance eosinophil and inactive fibroblast (in µm) | 14.5 (14.2;34.2) | 145 (88.7;203.3) | 47.6 (19.1;72.6) | 117.3 (79.8;117.5) | 29.7 (12.9;131.7) | 70.3 (41.2;187.9) | 18.4 (13.6;31.9) | 103.4 (82.5;177.8) |
| Distance eosinophil and active fibroblast (in µm) | 28.9 (8.7;98.3) | 163.2 (61.6;174.5) | 30.9 (18.3;35.3) | 61.4 (56.1;205.6) | 16.7 (13.4;17.9) | 138.8 (118.4;141.2) | 27.7 (20.0;86.0) | 48.3 (20.0;74.2) |


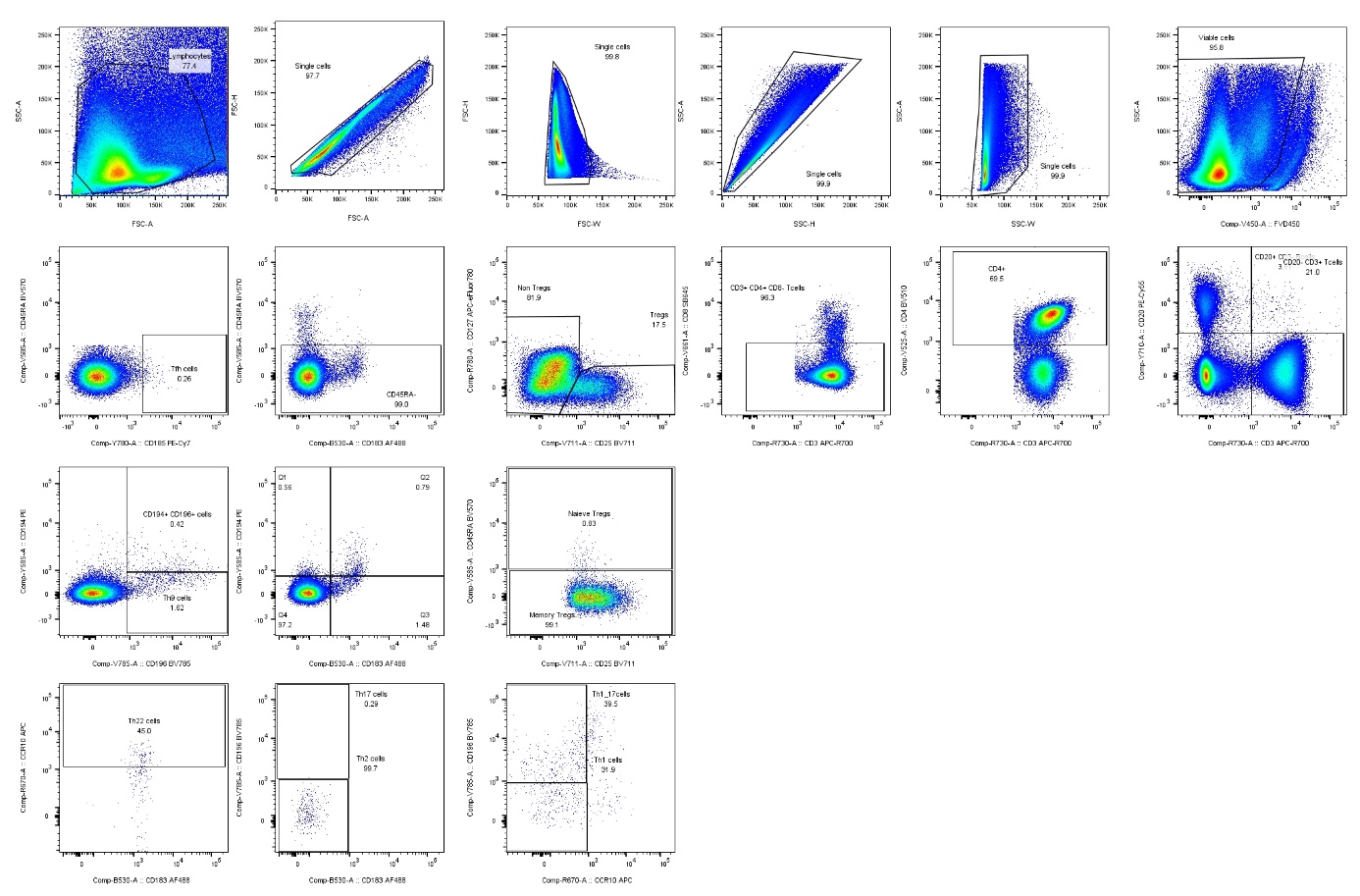


T cells

B cells

**Supplementary figure 1**: Gating adaptive panel

Via this panel, based on OMIP-17, we identified B cells (live CD3- CD20+ cells), T cells (live CD3+ CD20- cells), regulatory T cells (live CD3+ CD20- CD4+ CD8- CD25+ CD127-), Naïve regulatory T cells (live CD3+ CD20- CD4+ CD8- CD25+ CD127- CD45RA+), memory regulatory T cells (live CD3+ CD20- CD4+ CD8- CD25+ CD127- CD45RA+), T_FH_ cells (live CD3+ CD20- CD4+ CD8- CD25- CD127- CD45RA- CD185+), Th9 (live CD3+ CD20- CD4+ CD8- CD25- CD127- CD45RA- CD196+ CD194-), Th22 (live CD3+ CD20- CD4+ CD8- CD25- CD127- CD45RA- CD196+ CD194+ CCR10+), Th17 (live CD3+ CD20- CD4+ CD8- CD25- CD127- CD45RA- CD183- CD194+ CD196+), Th2 (live CD3+ CD20- CD4+ CD8- CD25- CD127- CD45RA- CD183- CD194+ CD196-), Th1 (live CD3+ CD20- CD4+ CD8- CD25- CD127- CD45RA-CD183+ CD194- CCR10- CD196-) and Th1_Th17 cells (live CD3+ CD20- CD4+ CD8- CD25- CD127- CD45RA- CD183+ CD194- CCR10- CD196+).


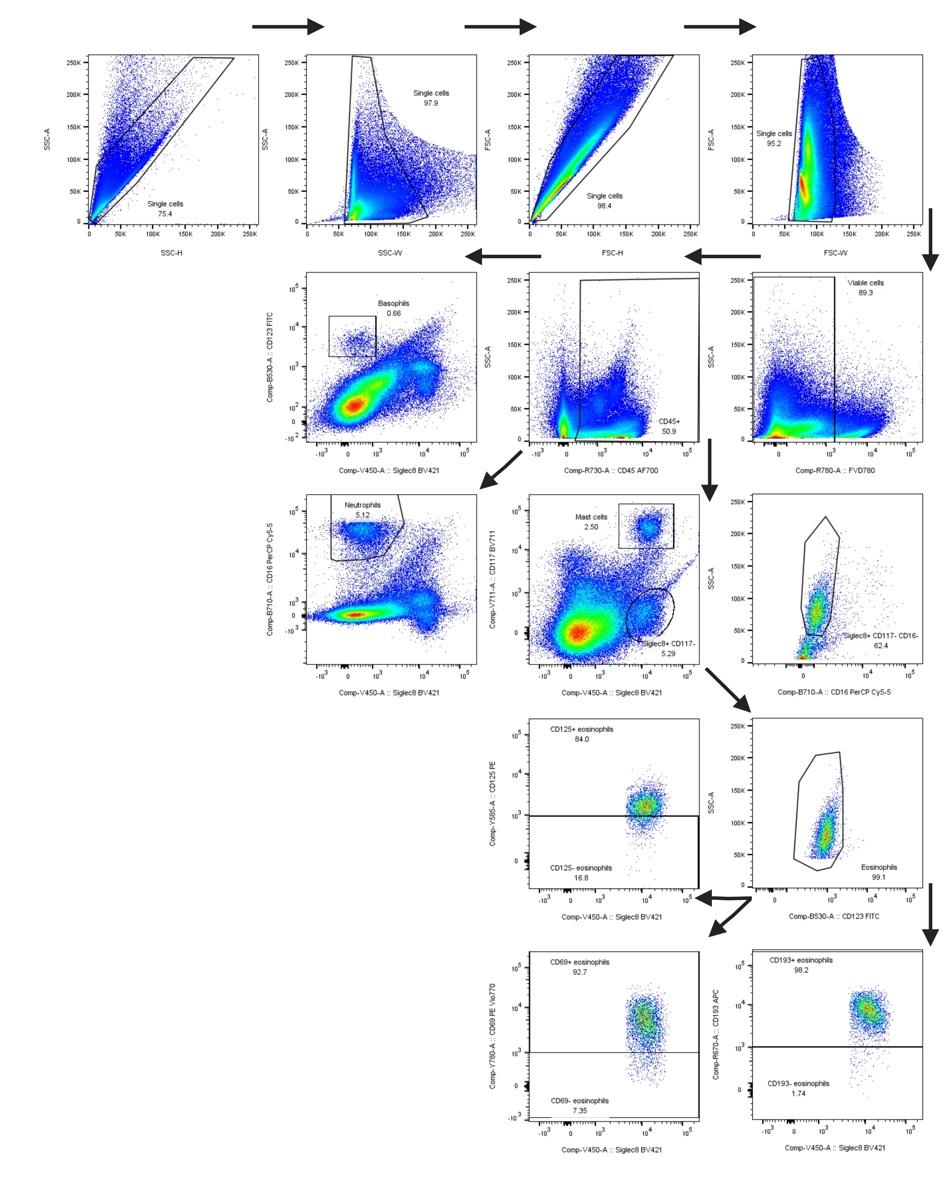


**Supplementary figure 2:** Gating eosinophil characterization panel

Eosinophils were identified as live CD45+ CD117- Siglec8+ CD16- CD123- cells after which CD193, CD125 and CD69 expression was assessed. Basophils were gated as live CD45+ Siglec8- CD123+ cells, mast cells as live CD45+ CD117+ Siglec8+ cells and lastly neutrophils as live CD45+ Siglec8- CD16 cells.


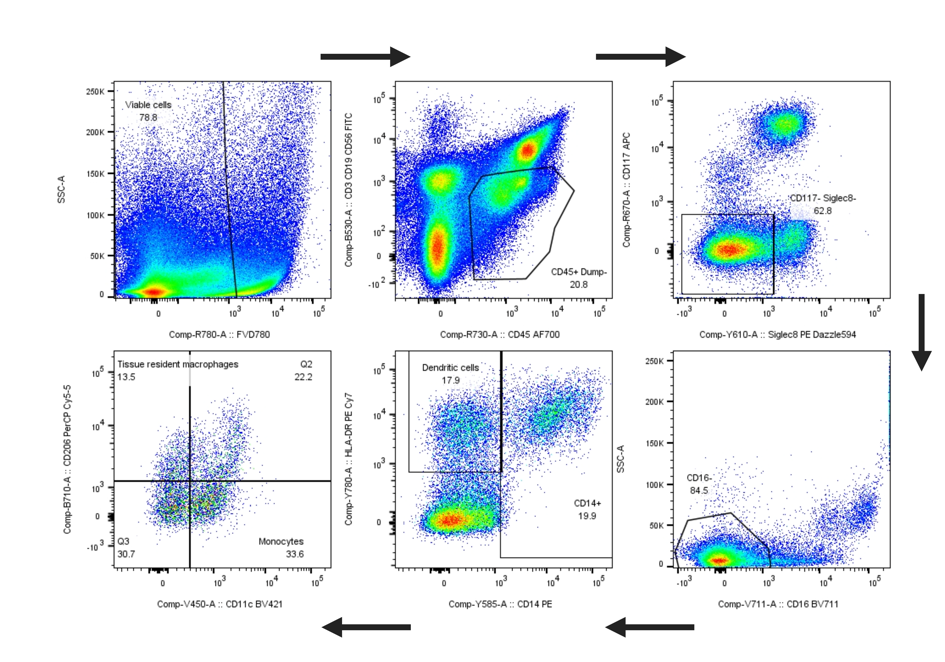


**Supplementary figure 3:** Gating innate panel

Via this panel we identified dendritic cells (live CD45+ CD3- CD19- CD56- CD117- Siglec8- CD16- CD14- HLA DR+), tissue resident macrophages (live CD45+ CD3- CD19- CD56- CD117- Siglec8- CD16- CD14+ CD11c- CD206+) and monocytes (live CD45+ CD3- CD19- CD56- CD117- Siglec8- CD16- CD14+ CD11c+ CD206-).
